# Effect of evidence-based education program with mentoring system for prevention of ventilator-associated pneumonia on nurses’ competency to improve quality care for patients in the intensive care unit: a quasi-experimental study in a tertiary hospital in Bangladesh

**DOI:** 10.3389/fpubh.2025.1674223

**Published:** 2026-01-09

**Authors:** Nahida Akhter, Xintong Zhou, Sameh Elhabashy, K. A. T. M. Ehsanul Huq, Miho Okamoto, Md. Abdul Latif, Abdulfatai Olamilekan Babaita, Michiko Moriyama

**Affiliations:** 1 Graduate School of Biomedical and Health Sciences, Hiroshima University, Hiroshima, Japan; 2 Faculty of Nursing, Cairo University, Cairo, Egypt; 3 Intensive Care Unit, Hiroshima University Hospital, Hiroshima, Japan; 4 Nursing Administration, The Project Capacity Building for Nursing Services Phase II (CBNS-II), Dhaka, Bangladesh

**Keywords:** evidence-based practice, intensive care units, mentoring, nursing staff, ventilator-associated pneumonia

## Abstract

**Background:**

Nurses’ skills, knowledge, practice, motivation, and work engagement are crucial for achieving better outcomes in intensive care unit (ICU) patients. Evidence-based in-service training and monitoring of nurses’ behavior can significantly enhance the quality of care for ICU patients. This study evaluates the effectiveness of an Evidence-Based Practice (EBP) training and mentoring system in improving ICU nurses’ competencies including skills, knowledge, clinical practices, motivation and work engagement for preventing ventilator-associated pneumonia (VAP) in a tertiary hospital in Bangladesh.

**Methods:**

A quasi-experimental pre- and post-intervention study was conducted from October 2024 to April 2025 in the ICU of Dhaka Medical College Hospital (DMCH) in Bangladesh. We provided a competency-based educational program to nurses who worked in the ICU, focused on EBP for VAP prevention, with mentoring support from trained mentors. Mentors were the nurses who received EBP training on VAP prevention from the research team prior to the intervention. Data were collected using a structured questionnaire. The changes in nurses’ skills, knowledge, practice, motivation and work engagement were assessed using validated scales before and after the EBP training and implementation of EBP. Higher scores of the scales indicate better outcomes.

**Results:**

The results showed significant improvements in nurses’ skills, knowledge, practice, motivation and work engagement following EBP training (all, *p* < 0.001). After the EBP training, 88.5% nurses achieved high skill scores, and 84.6% maintained high performance throughout the implementation period. Significant correlations were observed between knowledge and skill (*r* = 0.294) after training; knowledge and practice (*r* = 0.335), motivation and work engagement (*r* = 0.320), and skill with motivation and work engagement (*r* = 0.275) (all, *p* < 0.05) after EBP implementation.

**Conclusion:**

A structured EBP training program and mentoring system significantly enhanced ICU nurses’ competency (skills, knowledge, practice, motivation and work engagement) in EBP. The study highlights the importance of professional development, ongoing monitoring, and motivational strategies to support behavioral changes among nurses in low-resource settings.

**Clinical trial registration:**

ClinicalTrials.gov, NCT06624540.

## 1 Introduction

Evidence-based practice (EBP) in nursing is a methodological approach for clinical decision-making that integrates the best available research findings, clinical experience and patients’ preferences (1). EBP helps nurses to apply the best research findings into their practice (2). It also includes the clinical knowledge gained through experience (3) and respects what patients want and need in their care (4). A recent study confirmed that EBP was essential in nursing to provide patients with safe, effective, and high-quality care (5). A scoping review found that 90% of studies reported better patient outcomes when EBP was implemented, and 94% showed a positive return on investment (6). In intensive care units (ICUs), EBP plays an important role in providing quality care, reducing errors, educating healthcare professionals, ensuring successful treatments and fostering trust among patients and their families (7).

Despite its benefits, EBP implementation remains limited in low- and middle-income countries (LMICs), especially within ICUs (8). Studies assessing nurses’ EBP-related competencies, primarily focusing on knowledge and skills, were limited in number and often lacked a rigorous research design (9). Evidence suggests that many ICU nurses in LMICs require ongoing education to improve patient outcomes and enhance care quality (10).

Ventilator-associated pneumonia (VAP) is one of the most common and serious adverse events in ICU patients worldwide, affecting 7 to 43% of mechanically ventilated patients (11). VAP increases prolonged hospital stays, healthcare costs, and mortality (12). In Bangladesh, national data on the ICU VAP rate is not available. However, local reports showed that the ICU mortality rate was about 57.3% and the estimated VAP incidence was as high as 90% at Dhaka Medical College Hospital (DMCH) in 2021 (Internal report, 2021).

There are several reasons why VAP rates are high in LMICs like Bangladesh. Rahman 2023 explained that EBP was difficult to implement as there were limited research resources, a lack of advanced clinical knowledge among health professionals, poor nursing leadership, and shortages of equipment (13). On the other hand, critical care settings in Bangladesh face numerous challenges, including low competency levels among nurses and inadequate monitoring systems (14). Other problems include practitioners not following care guidelines (15), not using advanced tools like closed suction systems (16), a shortage of trained nurses (17), situations where family members provide ICU care (18) and poor institutional hygiene systems (19). Barriers such as limited nurse in-service training, ineffective mentoring, and weak hospital support further hinder adherence to standard guidelines (20).

To improve ICU care quality, it is essential to enhance nurse clinical competencies and establish effective monitoring mechanisms (21). Strengthening EBP requires targeted efforts to improve nurses’ competency (skills, knowledge, clinical practices, motivation and work engagement) (22, 23). From a Bangladesh perspective, identifying gaps and implementing appropriate interventions and monitoring systems are crucial steps toward improving critical care (24, 25). These insights can inform the development of effective educational programs and support systems for ICU nurses (26).

We adopted the Advancing Research and Clinical Practice through Close Collaboration (ARCC) model to identify barriers, develop EBP mentoring systems, and implement supportive strategies for daily clinical practice (27).

Therefore, this study aimed to evaluate the effectiveness of an EBP training and mentoring system in improving ICU nurses’ competency (skills, knowledge, practices, motivation and work engagement) for preventing VAP in a tertiary hospital in Bangladesh.

## 2 Methods

### 2.1 Study design and setting

A pre- and post-quasi-experimental study was conducted from October 2024 to April 2025 in a 32-bed ICU at DMCH, a leading tertiary-level government hospital located in the capital city of Bangladesh. DMCH is a 2,600-bed facility and houses nine distinct ICUs, accommodating a diverse range of patients across its 145 ICU beds, staffed by 202 nurses. Due to the rotational assignment of nurses across different ICU units, establishing a control group within these settings was not feasible.

### 2.2 Framework of this study

**Figure 1:**
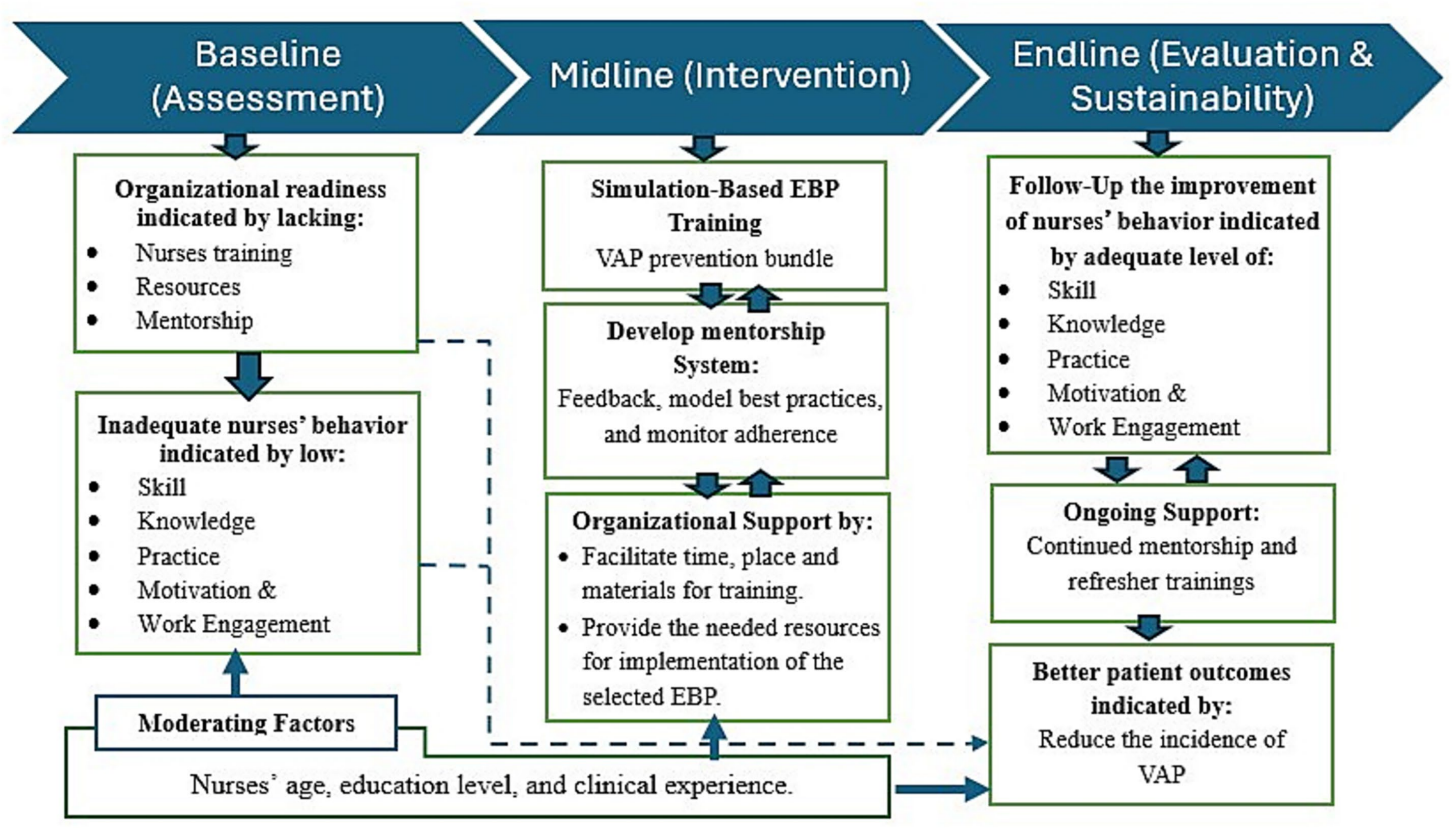
Research framework of this study.

This study framework is illustrated in Figure 1. The baseline assessment indicated nurse competencies including skills, knowledge, practice, motivation and work engagement, depended on organizational readiness in terms of training, resources and mentorship. The intervention phase implements three key strategies: simulation-based EBP training focused on VAP prevention, a structured mentorship system for guidance and monitoring, and organizational support that can initiate better patient outcomes.

### 2.3 Participants

All registered nurses *n* = 54 working in the General ICU were included in the study. The general ICU deals with patients’ life-threatening medical, surgical and trauma conditions. Eligibility criteria required nurses to be willing to participate in the EBP training and to provide informed consent. Nurses who were not directly involved in patient care within the ICU were excluded from the study.

### 2.4 Sample size

The sample size was determined using G*Power software version 3.1.9.4, Psychonomic Society, Madison, WI, USA, based on parameters from a previous study (28). The calculation assumed a two-tailed test with an alpha level of 0.05, statistical power of 0.95, an effect size of 0.56, and a confidence level of 95%. Accounting for an anticipated 5% dropout rate, the required sample size was estimated to be 52. However, due to the nature of the study, all eligible nurses were included.

### 2.5 Outcomes

Our primary outcome was the nurses’ VAP management skill measured by the skill performance checklist. Secondary outcomes were the nurses’ knowledge, practice, motivation and work engagement related to EBP.

### 2.6 Measurements

#### 2.6.1 Skill performance checklist

A researcher-developed skill assessment tool based on internationally recognized guidelines, including those from the American Association of Critical-Care Nurses, the American Association for Respiratory Care (AARC) (29), and the National Institutes of Health (NIH) (30). Additionally, two validated checklists were incorporated: the Closed Tracheal Suction Skill Checklist (31) and the Evidence-Based Nursing Competency Assessment Checklist (EBNCAC) (32).

Content validity was established using Lynn’s method (33). Three certified critical care nurses independently evaluated each item for relevance, clarity, and appropriateness of the grading scale. Based on their feedback, necessary revisions were made. The final version of the checklist consisted of 64 items related to VAP care, with a total maximum score of 140. Performance levels were operationally categorized as high ≥112/140 score; ≥80%, moderate 70–111/140 score; 50–79%, and low ≤69/140 score; <50% (32). Each item was scored as follows: “not performed” = 0, “unsatisfactorily performed” = 1, and “satisfactorily performed” = 2.

The item-level content validity index (I-CVI) was calculated, and the scale-level content validity index based on universal agreement S-CVI/UA was 0.97, indicating excellent content validity (33). Internal consistency reliability, assessed using Cronbach’s alpha, yielded a coefficient of 0.84, reflecting acceptable reliability.

The construct validity of the checklist was further checked and confirmed by three ICU specialist nurses at Hiroshima University Hospital, Japan. They reviewed each item to determine whether items adequately reflected the underlying construct, provided recommendations for revisions and reached consensus on the checklist. The researchers finalized the checklist based on their revision and recommendation.

#### 2.6.2 Knowledge scale

A researcher-developed tool was used to assess nurses’ knowledge related to VAP prevention. The scale consisted of 12 items, initially validated through a pre-test to evaluate construct validity. The same questionnaire was administered in the post-test phase. Each correct response was counted as one point, while incorrect responses received zero points. The development of the tool was informed by existing literature and validated sources, including Labeau et al. (34), Aysegul et al. (35), and Kapucu and Ozden (36).

#### 2.6.3 Practice scale

This was a researcher-developed scale designed to assess nurses’ practice related to VAP prevention based on previous studies (35, 37). The scale consisted of 6 items rated on a 5-point Likert scale, where 0 indicated “never” and 4 indicated “always.” Higher scores reflected better adherence to recommended practices. The content of the scale was reviewed and validated by certified critical care nurses from Hiroshima University Hospital.

#### 2.6.4 Motivation and work engagement scale

This scale was adapted from Karaferis et al. (38) and demonstrated acceptable internal consistency with a Cronbach’s alpha of 0.70. It comprised 12 items, each rated on a scale from 1 to 10, where 1 indicated the lowest level of motivation and 10 indicated the highest. The total score ranged from 12 to 120, with higher scores reflecting greater motivation.

### 2.7 Study procedures

#### 2.7.1 Prepare for mentorship

Following approval from the hospital and nursing directors at DMCH, 10 mentors from DMCH were recruited, who had active professional nursing licenses issued by the Bangladesh Nursing Council and a minimum of 2 years of clinical experience in areas such as ICU care or infection control. Additional selection criteria included demonstrated clinical management capabilities, proficiency in information technology, effective communication skills within multidisciplinary teams, and fluency in English, as the consent forms and questionnaires were administered in English.

The mentors were responsible for delivering evidence-based education, conducting bedside teaching in the ICU, and monitoring the participating ICU nurses throughout the intervention. They ensured adherence to the evidence-based protocols, gathered feedback from nurses regarding challenges encountered during patient care, and supported continuous learning. Mentors played a crucial role in facilitating behavioral change and promoting sustained engagement with the intervention throughout the study period.

#### 2.7.2 EBP training for mentors

The research team, consisting of the principal investigator (PI), one researcher and one certified ICU specialist nurse, developed a 1-week training manual for mentors, which included lectures and simulation-based sessions aimed at enhancing their knowledge and clinical skills. To further standardize the training program, a skill-based video was produced by the research team and shown during the training session. This video was also provided for practice at home. Throughout the training period, the researchers collected regular feedback from the mentors to continuously improve and refine the structure and content of the training program.

The objective of the training was to build mentors’ capacity and equip them with updated EBP knowledge to support advanced clinical practice. The instructional video was later shared with the study nurses to reinforce learning and ensure consistency in the delivery of EBP interventions.

#### 2.7.3 Study nurses’ intervention procedures

PI and mentors obtained written informed consent from the study nurses. In the first 2 months, they collected data from the study nurses without any intervention. After that, they provided a two-month-long EBP training intervention to the study nurses. Intervention was provided by integrating EBP related to management of VAP, clinical decision-making, and improving nurses’ EBP concepts and practical knowledge. After EBP training, nurses implemented their learning experiences at the patient’s bedside. Mentors observed the nurses’ patient care data analysis related to VAP and evaluated their acquired skills, knowledge, practice, motivation and work engagement improvement using different scales and checklists.

#### 2.7.4 EBP training for the study nurses

The PI, a doctoral student specializing in intensive care and the first author, along with the mentors, conducted weekly training sessions for the study nurses over a period of 1 month. We set a weekly training for 1 month. We utilized multi-model training sessions including lectures, virtual reality (VR) and simulation-based active learning. The training was designed to enhance nurses’ cognitive behavior and psychomotor change. Each of the four sessions focused on the prevention of VAP in the ICU (Table 1).

**Table 1:** Component of the evidence-based education program.

Session	Lecture	VR training	Skill training by simulator
1	Introduction to quality indicator and EBP Current status comparison with developed and developing countries.	–	Access to evidence-based guidelines
2	Introduction to VAP bundle- hand Hygiene, oral care, ulcer and DVT prophylaxis	Oral care	Skill training of bundle components-oral care, hand hygiene and DVT measurement
3	VAP bundle (continue)-patient position, endotracheal suction	Endotracheal suction	Skill training of bundle components- patient. position and endotracheal suction
4	VAP bundle and Prevention of VAP- weaning, ventilator circuit change, early ambulation Responsibility of patient care and activities	–	Skill training bundle of components- weaning, early ambulation and ventilator circuit change followed by case studies and simulation.

The education session was structured and organized combined with a 3-h lecture and 2 h of simulation-based training. Initially, the researcher introduced EBP concepts to the study nurses and stimulated nurses’ awareness by comparing with Japan.

To contextualize the importance of VAP prevention, the researcher presented data on ICU-related adverse events and statistics specific to VAP. The instructional content was organized into three lectures, each focusing on components of the VAP prevention bundle, which was developed in accordance with the Centers for Disease Control and Prevention (CDC) guidelines.

The first lecture aimed to help nurses construct an image of VAP prevention procedures, which was reinforced through the use of VR and a visual demonstration video produced in Japan. This visualization phase enabled the nurses to transition effectively into simulation-based practice. During the simulation training, participants engaged in case studies and scenario-based exercises, allowing them to apply theoretical knowledge in a controlled, practical environment.

### 2.8 Data analysis

Data were analyzed using SPSS version 30.0 (IBM Corp., Armonk, NY, USA). Continuous variables were assessed for normality and summarized using means and medians accordingly. Categorical variables were presented as frequencies and percentages. Comparing outcomes across three time points (before and after training, and implementation periods), Friedman test and Wilcoxon signed ranks test were conducted. A multiple regression analysis was employed to examine the association between dependent variable (nurses’ skills, knowledge, practice, motivation and work engagement) and independent variables including age, educational attainment, total work experience, and ICU work experience. Correlations among the four dependent variables were also analyzed. A *p*-value of < 0.05 was considered statistically significant.

## 3 Results

All 54 nurses employed at the time of study enrollment were assessed for eligibility and subsequently enrolled. During the baseline data collection period, 2 nurses were excluded for transferring to another hospital. The remaining 52 nurses completed the EBP training intervention and clinical implementation phases and data were analyzed (Supplementary Figure 1).

### 3.1 Sociodemographic characteristics of participants

All 52 nurses were female and held the position of senior staff nurse. The mean age (SD) was 33.4 (6.2) years. Regarding educational qualifications, 40.4% held a diploma, 38.5% had a Bachelor of Science in Nursing (BSc), and 21.2% possessed a Master of Public Health (MPH) or Master of Science in Nursing (MSN). The mean (SD) total work experience was 10.4 (5.8) years and within this time, they worked in the ICU 7.6 (5.0) years (Table 2).

**Table 2:** Sociodemographic characteristics of participants, *n* = 52.

Variables	Mean (SD), *N* (%)	Median^#^	Min	Max
Age	33.4 (6.2)	32	26	54
Education level				
Diploma	21 (40.4%)	–		
BSc in Nursing	20 (38.5%)	–		
MPH/MSN	11 (21.2%)	–		
Total work experience years	10.4 (5.8)	8.5	3	35
Work experience in ICU years	7.6 (5.0)	6.5	2	25

^**#**^As the data were left-skewed, we reported median. MPH, Master of Public Health; MSN, Master of Science in Nursing; SD, standard deviation; ICU, Intensive Care Unit.

### 3.2 Improvement of nurses’ skills, knowledge, practice, motivation and work engagement

Table 3 presents a comparison of skills, knowledge, practice, motivation and work engagement scores measured at three time points: before training, after training and implementation periods. All 4 items showed statistically significant improvement immediately after the training (all, *p* < 0.001). Scores of skill components improved uniformly (all, *p* < 0.001), and were maintained or slightly increased during the implementation period.

**Table 3:** Comparison of nurses’ skill, knowledge, practice, motivation and work engagement scores across three time points.

Dependent variables	Mean (SD)			Friedman test		Wilcoxon signed ranks test					
	Before training	After training	Implementation phase	χ^2^	*p*-value	*^#^Z* ^1^	*p*1	*^##^Z* ^2^	*p*2	*^###^Z* ^3^	*p*3
Skill (Overall)	31.7 (12.8)	120.9 (7.4)	121.4 (9.4)	78.3	<0.001	−6.3	<0.001	−0.4	0.709	−6.3	<0.001
Hand Hygiene	3.1 (1.2)	9.7 (0.7)	9.5 (1.0)	90.2	<0.001	−6.3	<0.001	−1.1	0.269	−6.3	<0.001
Oral care	5.8 (1.7)	19.1 (2.5)	19.8 (2.3)	83.7	<0.001	−6.9	<0.001	−1.7	0.083	−6.3	<0.001
Patient Position	3.7 (1.9)	15.0 (2.0)	13.0 (1.4)	92.2	<0.001	−6.3	<0.001	−5.1	<0.001	−6.3	<0.001
Endotracheal tube Suction	7.1 (4.0)	39.8 (3.9)	39.2 (2.7)	82.3	<0.001	−6.3	<0.001	−1.4	0.150	−6.3	<0.001
Circuit change	2.7 (1.5)	7.8 (1.3)	8.9 (1.3)	81.9	<0.001	−6.2	<0.001	−3.6	<0.001	−6.2	<0.001
Early ambulation	3.7 (3.1)	9.5 (1.8)	10.7 (2.6)	64.5	<0.001	−6.1	<0.001	−2.4	0.015	−6.0	<0.001
Weaning	2.3 (1.7)	4.9 (1.3)	5.6 (0.8)	67.1	<0.001	−5.1	<0.001	−2.8	0.006	−5.9	<0.001
Ulcer and DVT Prophylaxis	3.2 (1.9)	15.2 (2.2)	14.5 (2.7)	82.1	<0.001	−6.3	<0.001	−1.5	0.124	−6.3	<0.001
Knowledge	6.0 (1.7)	9.1 (1.5)	10.6 (1.5)	82.2	<0.001	−5.9	<0.001	−5.3	<0.001	−6.2	<0.001
Practice	16.2 (3.7)	21.9 (1.4)	22.4 (1.0)	82.4	<0.001	−6.3	<0.001	−2.1	0.035	−6.3	<0.001
Motivation and work engagement	35.6 (21.5)	71.7 (27.6)	78.0 (22.2)	52.3	<0.001	−5.5	<0.001	−1.3	0.210	−6.0	<0.001

^#^Z^1^-Before and after training. ^##^Z^2^- After training and implementation phase. ^###^Z^3^- Before training and implementation phase. SD- standard deviation, χ2- Chi-Square test, Z-standardized test statistic from Wilcoxon test. Score range: Skill 0–140, Knowledge 0–12, Practice 0–24, Motivation and work engagement 12–120.

**Figure 2:**
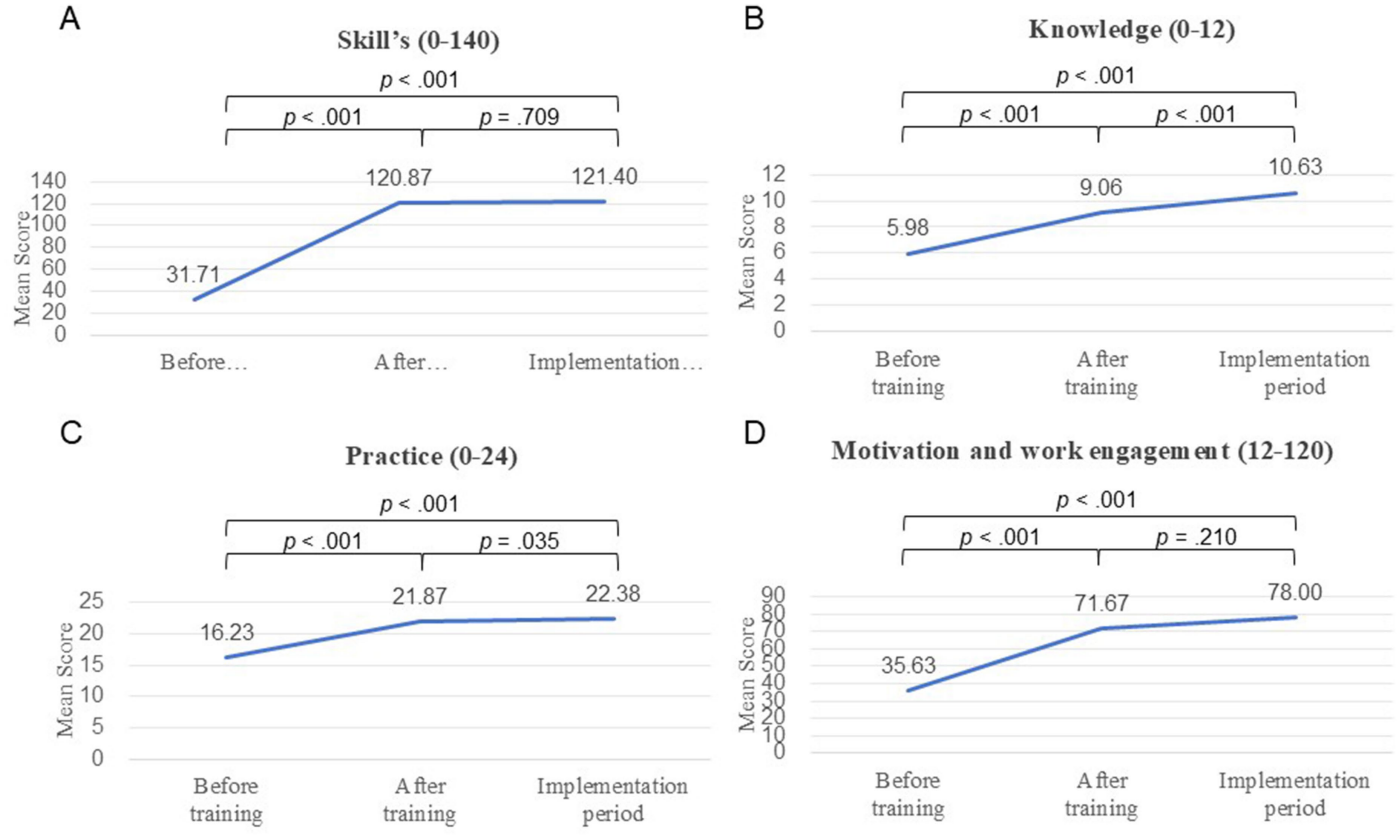
Mean difference of **(A)** skill, **(B)** knowledge, **(C)** practice, **(D)** motivation and work engagement score.

Among all 8 skill components, 4 of them, namely: patient positioning, circuit changes, early ambulation, and weaning demonstrated statistically significant improvements both after the training and the implementation period (all, *p* < 0.05). The overall skill (*p* = 0.709) and some of its components (hand hygiene; *p* = 0.269, oral care; *p* = 0.083; endotracheal tube suction; *p* = 0.150, ulcer and DVT prophylaxis; *p* = 0.124) and motivation and work engagement (*p* = 0.210) were not improved during implementation phase after training. However, none of the skill scores reached the maximum possible score of 140. The mean differences in skills, knowledge, practice, motivation and work engagement across the study phases are presented in Figure 2.

**Figure 3:**
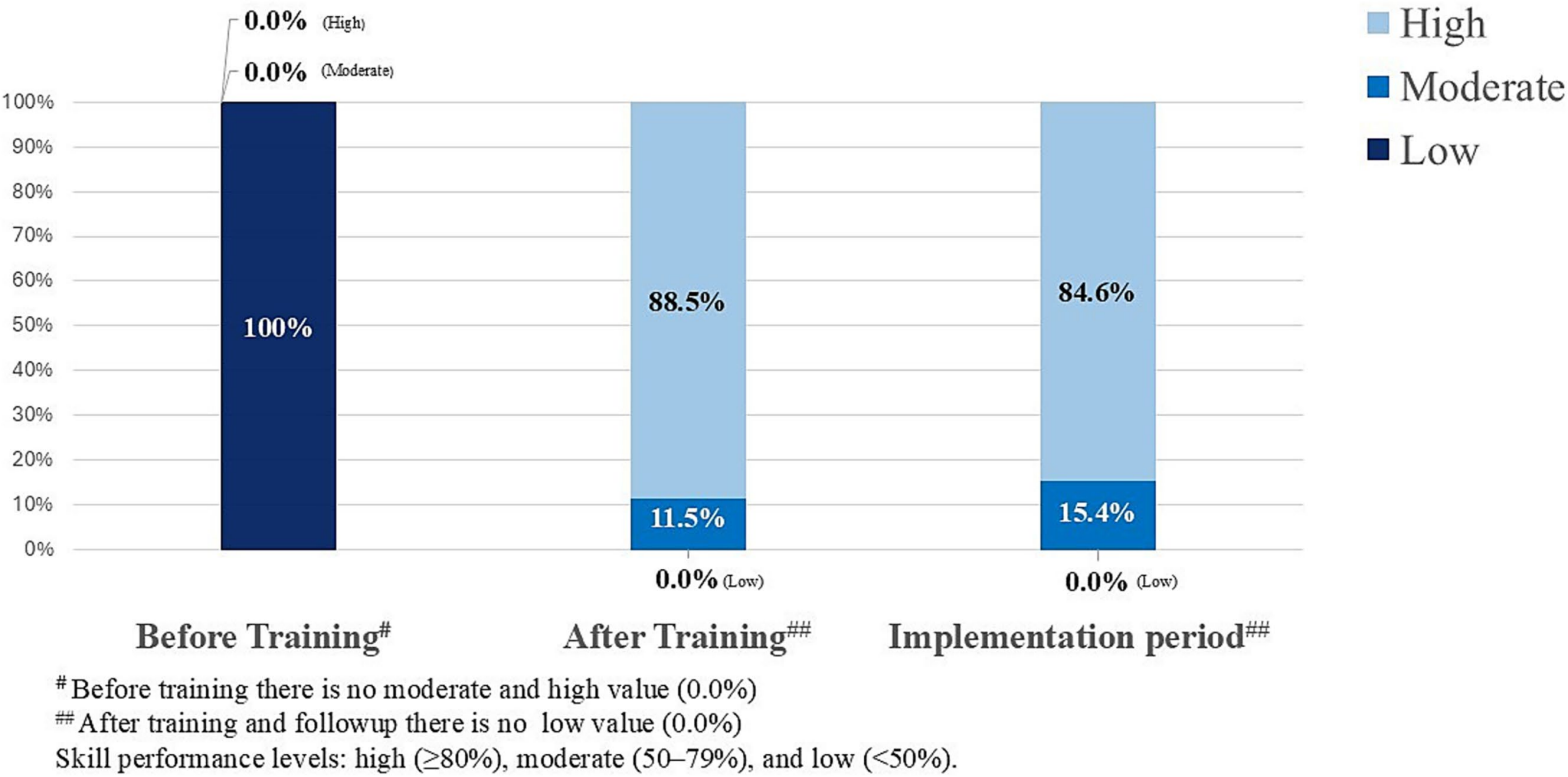
Bar diagram of total skill score.

As illustrated in Figure 3, in which the skill scores were re-categorized into three levels: high, moderate and low scores, prior to the training, 100% of participants scored in the low category. Following the training, 88.5% achieved high score, and at the assessment during the implementation period, 84.6% maintained high performance level. Knowledge and practice scores continued to improve significantly throughout the study period (all, *p* < 0.001). Motivation and work engagement showed borderline statistical significance at the implementation period, with mean scores reaching approximately 65% of the maximum.

### 3.3 Examining the effect of socio demographic factors on skill, knowledge, practice, motivation and work engagement

In the multiple regression analyses, at baseline, both age and educational level were significantly associated with skill scores. Specifically, older age was positively correlated with higher skill scores (*B* = 0.582, *p* = 0.023), and higher educational attainment was also a significant predictor (*B* = 6.264, *p* = 0.003). However, these professional backgrounds did not show a significant effect on knowledge, practice, motivation and work engagement. Longer total work experience was negatively associated with motivation and work engagement (*B* = −1.938, *p* = 0.046). Following the training intervention and implementation phases, the effects of age, education, work experience, and ICU experience on all measured components were no longer statistically significant (Table 4).

**Table 4:** Multiple regression analysis examining the effects of socio-demographic factors.

Dependent variables	Age		Education level		Work experience		ICU work experience	
	*B*	*p*	*B*	*p*	*B*	*p*	*B*	*P*
Before training								
Skills	0.582	0.023	6.264	0.003	0.083	0.867	0.861	0.144
Knowledge	−0.004	0.920	0.304	0.348	−0.010	0.898	−0.068	0.467
Practice	0.093	0.282	−0.603	0.384	−0.138	0.424	0.135	0.501
Motivation and work engagement	−0.033	0.945	−3.812	0.322	−1.938	0.046	1.106	0.322
After training								
Skills	−0.038	0.829	0.254	0.857	−0.095	0.787	−0.101	0.806
Knowledge	−0.044	0.188	0.412	0.124	0.084	0.206	−0.055	0.476
Practice	0.022	0.502	−0.018	0.945	−0.026	0.701	0.053	0.495
Motivation and work engagement	0.606	0.339	0.012	0.998	0.209	0.166	−1.529	0.304
Implementation phase								
Skills	−0.075	0.735	1.648	0.356	0.218	0.623	−0.012	0.981
Knowledge	−0.040	0.269	0.117	0.685	−0.003	0.962	0.058	0.491
Practice	−0.039	0.103	−0.071	0.711	−0.027	0.576	0.032	0.565
Motivation and work engagement	0.073	0.890	−2.010	0.636	0.655	0.536	−0.544	0.659

B, unstandardized regression coefficient, p < 0.05. ICU, intensive care unit; DVT, deep vein thrombosis.

### 3.4 Correlation among knowledge, practice, skill, motivation and work engagement

To explore the interrelationships among skill, knowledge, practice, motivation and work engagement, correlation analyses were conducted at three key time points. Before the training, a weak positive correlation was observed between knowledge and practice (*r* = 0.244, ns), though this relationship was not statistically significant. After the training, a statistically significant but weak correlation emerged between skill and knowledge (*r* = 0.294, *p* < 0.05), indicating a modest alignment between theoretical understanding and practical ability.

During the implementation phase, a weak yet statistically significant correlation was found between skill and motivation/work engagement (*r* = 0.275, *p* < 0.05). Additionally, knowledge showed moderate, statistically significant correlations with both practice (*r* = 0.335, *p* < 0.05) and motivation/work engagement (*r* = 0.320, *p* < 0.05) (Table 5).

**Table 5:** Pearson correlation among nurses’ skill, knowledge, practice, motivation and work engagement scores across time points.

Correlations		Skill	Knowledge	Practice	Motivation and work engagement
Before EBP training	Skills	–	–	–	–
	Knowledge	0.018	–	–	–
	Practice	0.077	0.244	–	–
	Motivation and work engagement	0.034	0.132	0.081	–
After EBP training	Skills	–	–	–	–
	Knowledge	0.294*	–	–	–
	Practice	0.075	−0.043	–	–
	Motivation and work engagement	0.069	0.100	0.155	–
Implementation phase	Skills	–	–	–	–
	Knowledge	−0.139	–	–	–
	Practice	−0.135	0.335*	–	–
	Motivation and work engagement	0.275*	0.320*	0.221	–

^*^Correlation is significant at the 0.05 level (two-tailed).

## 4 Discussion

To the best of our knowledge, this is the first study in Bangladesh to assess ICU nurses’ knowledge, skills, practices, motivation and work engagement related to VAP prevention. This study implemented an EBP education program with a mentoring system to evaluate nurses’ competencies including skills, knowledge, clinical practice, motivation and professional engagement for VAP prevention among the ICU patients and observed significant improvements in these outcomes. The EBP education was delivered through a combination of lectures, VR-based image training, simulation, and hands-on practice, and the ARCC model had a profound impact in a short time. Following the training, nurses applied their knowledge directly to ICU patient care. This study tried to identify the level of competency of ICU nurses and found a lack of motivation and work engagement, which are essential for sustainable practice change. The findings offer important insights into the feasibility and potential impact of EBP interventions in resource-constrained critical care settings.

The skill-based competency after providing EBP training and implementation of intervention was evaluated and found overall skill scores were significantly increased among the nurses. We found that overall skill scores were increased immediately after training, demonstrating that theoretical instruction combined with simulation and mentoring can lead to rapid gains in knowledge and confidence. This finding aligns with previous studies reporting similar improvements following EBP training (32). However, skill scores did not increase further during the implementation phase, when nurses began applying what they learned in a real ICU situation. This indicates that nurses’ skills improved theoretically within a short time; however, they need more time and continued support in patient care to develop their skills in clinical settings. To get a sustained improvement in skill, it needs long-term education, monitoring and motivation among nurses. This finding aligns with previous studies that have demonstrated the effectiveness of 5-day EBP training in enhancing clinical skills and underscores the importance of structured educational programs in improving patient care outcomes (39).

The component of skills related to patient positioning, ventilator circuit changes, and the weaning process showed marked improvement. These improvements likely reflect areas where nurses had direct opportunities to practice in the ICU, reinforcing the idea that practical repetition is essential for developing clinical competence. Therefore, while the EBP training was effective in building foundational skills, sustained mentorship and on-the-job reinforcement are critical to fully translating these competencies into high-quality patient care. These findings are consistent with previous research (40) and emphasize the importance of integrating practical learning strategies alongside didactic training in a critical care setting.

The skill-based competency was not associated with nurses’ age, level of education and work experience. This suggests that skill-based improvements were primarily driven by the intervention itself, rather than professional background, highlighting the value of accessible and standardized training for all nurses regardless of sociodemographic characteristics. However, consistent with prior literature, sustained improvement in clinical skills requires ongoing support systems (41), including supervision (42), sufficient resources (43), and manageable workloads (44). In low-resource ICU settings, challenges such as high patient loads, lack of clinical oversight, and limited equipment may hinder nurses’ ability to consistently apply newly acquired skills in practice.

Mentoring played a key role in the success of this intervention and was essential for supporting the use of EBP in ICU nursing. The structured mentoring system helped nurses develop their skills, apply what they learned in training, and gain confidence in using EBP in real patient care. This support was especially important in the ICU setting, where the work is fast-paced and stressful. Our findings suggest that mentoring does not just help with initial skill development; it also plays a crucial role in maintaining and reinforcing those skills over time. This highlights the importance of ongoing professional development, regular feedback, and supervision to keep the benefits of training in place. Providing mentorship through both the training and implementation phases, along with ensuring that nurses have the necessary resources, is key to making EBP a sustainable part of daily nursing care. These results are supported by earlier studies that show how mentoring improves nurses’ performance and contributes to better patient outcomes (45).

Nurses’ competency (skill, knowledge, practice, motivation and work engagement) was improved after providing EBP training and implementation of intervention to manage the ICU VAP patients. This aligns with previous literature emphasizing the role of structured educational interventions in improving clinical knowledge (46). Though their knowledge and practice scores increased from theoretical to practical implementation, the scores of motivation and work engagement did not increase during this period. This might need a longer monitoring system with additional on-the-job training, evaluation, and organizational acknowledgement (47) to increase motivation and work engagement (48). The improvement of practice scores depends on when knowledge acquisition translates into practice, supported by hands-on training and practical reinforcement.

The study demonstrated a substantial increase in nurses’ motivation and work engagement following the EBP training, with post-training scores reaching about 65%. However, during the implementation phase, these scores did not continue to improve, suggesting that training alone may not be enough to sustain engagement. This plateau likely reflects ongoing systemic challenges within ICU environments, such as high patient loads, limited autonomy, and a lack of organizational recognition (49). While nurses have become more invested in their roles, there remain considerable opportunities for improvement. Given that engagement is a key factor in sustaining EBP, future interventions should include not only technical training but also strategies to foster a positive work culture. These may include staff recognition, peer collaboration, nurse-led decision-making, and active leadership involvement (50). Supporting these elements can help maintain motivation and promote lasting practice change.

Sociodemographic factors such as age and education level were linked to differences in knowledge and practice scores before the training. However, these differences disappeared after the EBP training and during the implementation period. This suggests that the training program helps reduce initial gaps in learning caused by background differences, allowing all nurses to benefit equally. Moreover, structured and well-designed EBP education can improve clinical competencies regardless of a nurse’s age, education, or experience. These findings highlight the value of inclusive training programs in promoting equal learning opportunities and ensuring a consistent standard of patient care among diverse backgrounds of nursing staff, especially valuable in LMICs settings. Similar results have been reported in previous studies, where EBP training helped standardize practice across different groups of healthcare workers (51).

Before the training, there was no significant relationship between skills, knowledge, practice, motivation or work engagement. After the training, weak and moderate but meaningful correlations appeared — for example, between skills and knowledge, skills and motivation, and knowledge and practice. These findings suggest that clinical performance is influenced by a mix of cognitive knowledge, behavioral practice/skills, and emotional motivation/engagement factors. The correlation between skills and motivation shows that hands-on training not only improves technical ability but may also increase nurses’ interest and involvement in their work (52). The link between knowledge and practice highlights the importance of gaining knowledge to change practice. To truly improve clinical behavior, training must be supported by follow-up coaching, teamwork, and a work environment that encourages the use of new skills (53). The findings show EBP training with mentoring effectively enhances ICU nurses’ competencies in VAP prevention. Sustaining will require regular ongoing supervision and an education system. Long-term adherence strategies and an in-service training system can help to improve nurses’ competencies.

### 4.1 Limitations

Due to lack of control group, this study employed a quasi-experimental design which limits the ability to establish definitive causal relationships. Additionally, the involvement of ten mentors in evaluating the nurses introduces observational bias, as participants may have been more alert or performed differently under direct observation.

## 5 Conclusion

The results demonstrate that EBP training significantly enhances the care of ventilated patients by improving nurses’ competencies including knowledge, practice, skills, and motivation in their clinical practice. To sustain these improvements, ongoing education and mentoring systems are essential. The findings advocate integrating EBP training into routine in-service education and highlight the need for systemic support to overcome barriers such as workload, staff shortages, and inadequate monitoring systems. Future studies should explore the socio-behavioral determinants of VAP prevention and factors influencing adherence to guidelines. Additionally, further research should explore long-term strategies for maintaining motivation and practice adherence in resource-constrained settings.

## Data Availability

The data used and analyzed in this study are available from the corresponding author on reasonable request.
