# Supplementary material for: Effect of evidence-based education program with mentoring system for prevention of ventilator-associated pneumonia on nurses’ competency to improve quality care for patients in the intensive care unit: a quasi-experimental study in a tertiary hospital in Bangladesh
Source: Front Public Health. 2026 Jan 9;13:1674223. doi: 10.3389/fpubh.2025.1674223 (PMC12827743; doi:10.3389/fpubh.2025.1674223)
Supplement: Supplementary file 1 [file Data_Sheet_1.PDF]

Questionnaire Skill Scale

A: Skill performance checklist for Mechanically ventilated patients (Followed by VAP bundle guideline)

S = Satisfactory U = Unsatisfactory NP = Not Perform

Bundle element	Nursing Intervention	S<br>2	U<br>1	NP<br>0
Hand Hygiene	(1) All healthcare providers and patient's family members should wash their hands at the following occasions:<br>1) Before entering the patient care zone<br>2) Before contacting the patient<br>3) After touching the patient's body fluids or secretions<br>4) After moving away from the patient<br>5) After departing from the patient care zone	2	1	0
	(2) Healthcare workers should wash hands before and after touching the respiratory circuit.	2		0
	(3) If the hands are not visibly soiled, the hands could be cleaned with an alcohol-based antiseptics preparation instead of tap water and soap.	2		0
	(4) If the hands are visibly soiled, the hands should be washed with water and soap.	2		0
	(5) A hand disinfector should be set at an easily accessible location on the patient's bedside.	2		0
	**S-follow every step very carefully U- not doing every step NP-not doing.**			
Oral care	1. Disinfect hands, wear gloves and appropriate PPE for universal precaution	2	1	0
	2. Identify the patient by name and check consciousness level.	2		0
	3. Perform an assessment of the need for oral suctioning	2	1	0

	4. Identify supplies needed. Suction device, toothbrush, mouth solution.	2	1	0
	5. Explain to the patient the purpose and method of oral care and obtain consent	2		0
	6. Brush teeth, gums, and tongue at least three times a day with a soft toothbrush	2	1	0
	7. Use of 0.12% oral chlorhexidine to rinse the oral cavity in every night and another time use normal water (per shift)	2	1	0
	8. Moister oral mucosa and lips every 2-4 h.	2	1	0
	9. Discard mouth brush, using items and gloves and sanitize hands	2	1	0
	10.Remove apron and eye guards (goggles) and perform hand sanitization	2		0
	11. Inform the patient to complete the procedure and record the procedure.	2		0
	**S-follow every step very carefully U- not doing every step NP-not doing**			
Positioning	2. Explain and get permission to the patient for this procedure.	2		0
	1. Check for changes in the patient's condition	2		0
	3. Head of bed elevation to 30°–45°	4	2	0
	4. Change position every 2 hours	4	2	0
	5. Keep extremities in physiologically neutral position using pillows or appropriate splint/ support devices as indicated.	4	2	0
	**S-follow every step very carefully U- not doing every step NP-not doing.**			
Endo Tracheal	1. Disinfect hands, wear gloves and appropriate PPE for universal precaution.	2	1	0

Tube Suctioning	2. Identify the patient by name and check conscious level before suctioning, assure the patient with aseptic technique	2		0
	3. Perform an assessment of the need for oral and ET tube suctioning	2	1	0
	4. Assess for need for tracheal suctioning. (Perform visual, palpation, and auscultation)"<br><br>a. Assess for cyanosis or pallor by checking the skin color, and visual inspection of respiratory parameters (respiratory pattern and efforts, intercostal and suprasternal retraction, shallow respiration)<br><br>b. Auscultate the lungs field with the use of stethoscope diaphragm in a systemic pattern and identified the lung sound.<br><br>c. Check the bedside monitor to check for deviations in the respiratory and hemodynamic parameters.<br><br>d. Check the Ventilator monitor for deviations suggestive of inadequate oxygenation or accumulation of secretion (e.g sawtooth pattern, increased peak airway pressure)	4	2	0
	5. Check the fixation status and depth of the tracheal tube	4	2	0
	6. Connect the connecting tube to the suction device and Apply pressure to allow use of the suction device.	2	1	0
	7. Adjust suction pressure to less than (20kpa or 150mmHg) and check for negative pressure by occluding the end of the connecting tube	2		0
	8. Connect the oral suction tube to the connecting tube and aspirate oral secretions.<br><br>*Did not apply negative pressure when inserting the oral suction tube into the mouth<br><br>*Moved the catheter along the gum line towards the pharynx in a circular motion<br><br>*Avoided deep insertion to the end of the tongue (prevent gagging and vomiting)	2	1	0
	9. Discard the oral suction tube and gloves	2		0

	*Removed the gloves by Glove-in-Glove or Bird-Beak technique			
	10. Disinfect hands and put on glove	2		0
	11. Subglottic suctioning:<br>• Check cuff pressure every 4 hours to maintain endotracheal cuff pressure 25 cm -30 cm H_2O<br>• avoid excessive endotracheal tube movement.<br>• Connect the suction tube above the cuff to the connecting tube	4	2	0
	12. Observe the color, consistency, and volume of secretions, as well as respiratory and circulatory dynamics, while suctioning.<br>• Visually observed the secretion to determine deviations (e.g., infection)<br>• Checked the bedside monitor and ventilator for changes	2	1	0
	13. Hyperoxygenation before suctioning (100%). Hyperoxygenation the patient before and after each subsequent pass of the catheter for at least 30 seconds, and before reconnection to the ventilator.	2	1	0
	14. Use of closed suction catheters	2	1	0
	15. Withdraw the catheter 1 to 2 cm, and apply intermittent suction while rotating and removing the catheter	2		0
	16. Remove gloves and sanitize hands.	2		0
	17. Check for changes in the patient's condition and synchronization with the ventilator<br>• Checked the monitor for ventilatory and hemodynamic recovery	2	1	0
	18. Inform the patient that the procedure has been completed	2		0
	19. Record the procedure	2		0
	**S-follow every step very carefully U- not doing every step NP-not doing.**			
	1. Check the patient connection	2		0

Ventilator circuit change	2. Ask help for patient repositioning	2		0
	3. Change the ventilator circuit if any visible or observe according to manufacture recommendation (7-14 days)	2	1	0
	4. Water droplets pooled in the circuit should be removed in an aseptic manner upon detection or before postural change of the patient.	2	1	0
	4. Position a patient with a lung segment uppermost, utilize pillow to maintain position	2	1	0
Early ambulation	1. Analgesia or sedation-			
	a. Assess the patient physical condition such as vital sign and conscious level.	2	1	0
	b. Check patients pre and post sedation status.	2	1	0
	c. Check all (doctor and nurse) document of last history of sedation.	2		0
	d. Give the analgesia or sedation as prescribed and apply the sedation vacation protocol as the doctor ordered.	2	1	0
	2. Mobilization activity			
	a. Assess the patient condition (Vital sign and sedation status)	2		0
	b. Implement sedation vacation to prepare patient for mobility	2		0
	c. Perform Mobility activities (20 mins/day) according to patients' capacity.<br>• Passive/active range of motion<br>• dangling at edge of bed	2	1	0
	**S-follow every step very carefully U- not doing every step NP-not doing.**			

Weaning	1. To assess the possibility of weaning once daily with the use of a spontaneous breathing trial (SBT)	2		0
	2. Assess the patient's physical condition such as vital signs and conscious level.	2		0
	3.SaO_2 greater than 90% on FiO_2 40% or less, PEEP 5 cm, H_2O, Temperature normal, Normal ABG value.	2	1	0
	**S-follow every step very carefully U- not doing every step NP-not doing.**			
Ulcer and DVT prophylaxis	1.DVT prophylaxis-			
	a. Disinfect hands, wear gloves and appropriate PPE for universal precaution.	2	1	0
	b. Identify the patient.<br>*By calling the name of the patient to initiate communication and get permission for examination.	2		0
	c. Assess for sign and symptoms	2		0
	d. Assess the patients risk factor (obesity, injury, dehydration etc)	2	1	0
	e. Measuring leg circumference (difference more than 3 cm)	2	1	0
	f. Administered the prescribed medication for deep-vein thrombosis prophylaxis.	2	1	0
	**S-all step done perfectly, U- mistake step, NP-all mistake**			
	2. Peptic ulcer disease (stress ulcer) prophylaxis-			
	a. Assess the patient physical condition (epigastric tenderness and abdominal distention)	2		0
	b. Check the patient dietary supplement and monitor any hemorrhagic loss.	2	1	0
	c. Check and administer the prescribed medication for peptic ulcer disease (stress ulcer) prophylaxis.	2	1	0

	**S-follow every step very carefully U- not doing every step NP-not doing.**			

Results of Assessment Total score of (/140):

Competency level:

High (112 / >80%) …….... Moderate (70-111 / 50-80%) …….. Low (69 / <50%)

Pre-assessment: ….- • Immediate post-assessment: …..- • 2-month post assessment: …..

Nurse code: __________________________Date: ______________

Sex: years of experience in ICU:

Assessor Name: __________________________Date: ______________

**Questionnaire B: Question about the Knowledge regarding prevention of VAP** (select one)		
B1	Selection of endotracheal intubation method-	☐ Orotracheal intubation method is recommended.<br>☐ Nasotracheal intubation is recommended.<br>☐ Both methods are recommended<br>☐ I do not know
B2	Mechanical ventilator circuit change-	☐ Change is recommended every 48 hours.<br>☐ Change is recommended every 72 hours.<br>☐ Change is recommended for each new patient (not routinely)<br>☐ I do not know.
B3	Humidifier use for-	☐ Heated humidifiers are recommended.<br>☐ Heat-moisture exchangers are recommended.<br>☐ Both are recommended.<br>☐ I do not know
B4	Humidifier change	☐ Change is recommended every 48 hours.<br>☐ Change is recommended every 72 hours.<br>☐ Change is recommended every week.<br>☐ I do not know
B5	Selection of the aspiration system	☐ An open aspiration system is recommended.<br>☐ A closed aspiration system is recommended.<br>☐ Both methods are recommended<br>☐ I do not know
B6	A nurse required to discard a suction catheter	☐ Several time<br>☐ Immediately after a single use<br>☐ One's a day.<br>☐ Until it is use for choice
B7	Frequency of ETT suctioning should be done to the patients?	☐ Every 2 hours<br>☐ Every shift<br>☐ Daily<br>☐ **As needed**

B8	Use of endotracheal tubes with extra lumen for the aspiration of subglottic secretions	☐ Use of these endotracheal tubes reduces the development of VAP.<br>☐ Use of these endotracheal tubes increases the development of VAP.<br>☐ Use of these endotracheal tubes does not affect the development of VAP.<br>☐ I do not know
B9	Patient position need for VAP prevention	☐ The supine position is recommended.<br>☐ Semi-recumbent position (30-45 degrees) is recommended.<br>☐ The patient position does not affect the development of VAP.<br>☐ I do not know.
B10	Early weaning of mechanical ventilator mode results?	☐ Reduce patient costs.<br>☐ Reduce risk for further infection.<br>☐ Reduce the risk for VAP.<br>☐ For patient safety
B11	Use of oral antiseptics with chlorhexidine for oral care	☐ Use of this solution reduces the development of VAP.<br>☐ Use of this solution increases the development of VAP.<br>☐ The effect of this solution on the development of VAP is not known.<br>☐ I do not know
B12	Overfeeding a ventilated patients may increase the risk aspiration leading to?	☐ Abdomen distended.<br>☐ Leading to death<br>☐ Patient discomfort<br>☐ Increase the risk for VAP
**Questionnaire C: Question about the Practice regarding prevention of VAP** (select one)		
C1	Please indicate your level of familiarity with Evidence-Based Practice (EBP) in the context	☐ Very unfamiliar<br>☐ Very familiar<br>☐ Neutral<br>☐ I don't know

	of Ventilator-Associated Pneumonia (VAP) prevention.	
C2	I participate in VAP prevention-related research or educational activities.	☐ Never<br>☐ Rarely<br>☐ Occasionally<br>☐ Always.
C3	I use checklists or protocols to ensure comprehensive adherence to VAP prevention measures.	☐ Never<br>☐ Rarely<br>☐ Occasionally<br>☐ Always.
C4	I consistently follow hand hygiene protocol protocols before and after patient contact	☐ Never<br>☐ Rarely<br>☐ Occasionally<br>☐ Always.
C5	I routinely assess and maintain endotracheal tube cuff pressure within the recommended range (20 cmH_2O)	☐ Never<br>☐ Rarely<br>☐ Occasionally<br>☐ Always.
C6	I collaborate with infection control teams to enhance VAP prevention efforts.	☐ Never<br>☐ Rarely<br>☐ Occasionally<br>☐ Always.

Questionnaire D:
Factors Influencing Motivation and Work Engagement of Healthcare Professionals

	**Instractic motivation factor**	**1**<br><br>Lower motivation	**2**	**3**	**4**	**5**	**6**	**7**	**8**	**9**	**10**<br><br>Higher motivation
**1.**	Incentives for additional effort & productivity improvement										
**2.**	Recognition of contribution & progress										
**3.**	Opportunities to take initiatives & exploit resources										
**4.**	Ability to participate in making important decisions										
**5.**	Ability to progress / promotion										
**6.**	Opportunities to develop new knowledge-skills										
	**Extrinsic motivating factors**										
**1.**	Staff training, seminars & training in new systems-technologies										
**2.**	Good supervision & support from senior executives										
**3.**	Appreciation and good reputation										
**4.**	Flexible working hours										
**5.**	Satisfactory fees										
**6.**	Permanence–Occupational Safety										
