# Supplementary figures and images for: Effect of evidence-based education program with mentoring system for prevention of ventilator-associated pneumonia on nurses’ competency to improve quality care for patients in the intensive care unit: a quasi-experimental study in a tertiary hospital in Bangladesh
Source: Front Public Health. 2026 Jan 9;13:1674223. doi: 10.3389/fpubh.2025.1674223 (PMC12827743; doi:10.3389/fpubh.2025.1674223)

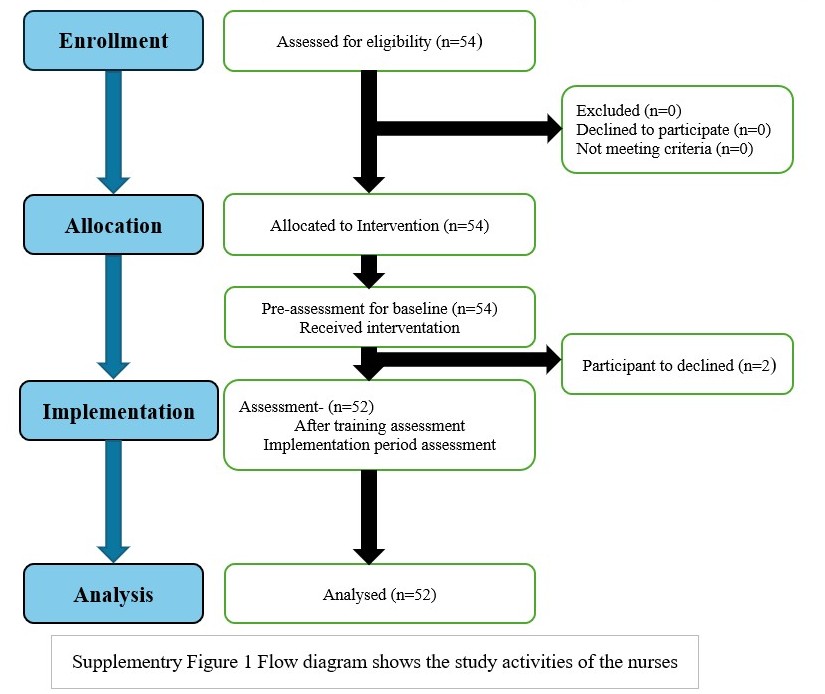

Supplement: Supplementary file 2 [file Image_1.JPEG]
